# Balancing public health and civil liberties in times of pandemic

**DOI:** 10.1057/s41271-020-00261-y

**Published:** 2021-01-18

**Authors:** Marcin Orzechowski, Maximilian Schochow, Florian Steger

**Affiliations:** Institute of the History, Philosophy and Ethics of Medicine, Ulm University, Parkstraße 11, 89073 Ulm, Germany

**Keywords:** Public health, Pandemics, Health policy, Ethics

## Abstract

The ongoing COVID-19 pandemic constitutes not only a danger for public health, but may also threaten civil liberties. Looking at the examples of recent events in Poland and Hungary, the authors argue that governments may misuse pandemic for their political advantage, thus endangering public health. Political decisions taken to stem the spread of pandemics should be limited and strictly proportionate to the situation.

## Introduction

The recent pandemic of the novel coronavirus SARS-CoV-2 poses a profound challenge, not only to public health systems around the globe, but also reaches deeply into very basic values on which many countries are built. As the global number of COVID-19 infection increases, political commentators and scientists are raising important questions: What does the spread of pandemic mean for democracies? How can we ethically balance public health and civil liberties?

The COVID-19 crisis presents an enormous spectrum of legal, ethical, and public health dilemmas. These range from allocation of scarce medical resources [1], equitable access to healthcare including medical care for vulnerable groups [2], intrusion into individuals’ private spheres through surveillance [3], decreasing free speech [4], and to questions of coercion in implementing rules about personal isolation and physical distancing [5]. The last three issues imply significant limitations of basic civil liberties. As the World Health Organization noted in 2007, enjoyment of individual human rights and civil liberties during pandemic emergency situations may have to be limited in the public interest; however, these measures need to be necessary, reasonable, proportional, equitable, non-discriminatory, and in full compliance with national and international laws [6]. A concern remains that governmental responses to the current pandemic may result in measures that will reach deeper than necessary and will outlast the Covid-19 crisis.

There is another view. An international mission of epidemiologists to China organized by the World Health Organization (WHO) stated in its report that the aggressive response imposed by the Chinese authoritarian regime led to containing the spread of the pandemic in that country [7]. If data provided by Chinese authorities are reliable, they show that the measures effectively stabilized the number of infections until an optimal level of societal immunity can be reached or until a vaccine is developed and in wide use in the future. In contrast, liberal democracies’ responses to the crisis seem inadequate. The number of infected people in the USA and several countries of Western Europe pushed the capabilities of their healthcare systems to the limit [8]. Anderson et al. express concerns that efforts to combat COVID-19 will be plagued by fake news and misinformation spread mostly through social media channels [9]. To counteract this, countries and social media companies need to act appropriately without undermining the right to free speech.

Given this situation, we highlight two important examples from Central Europe: Hungary and Poland. In Hungary, as of 7 September 2020, there were 8963 confirmed cases and 625 deaths caused by COVID-19. In Poland, the corresponding numbers are 71,126 and 2124, respectively [10]. Epidemiologists, political commentators, and media in both countries worry that the figures are deflated by low rates of testing [11]. Hungary has conducted less than a quarter of the number of tests than neighboring Austria, which has a slightly smaller population [12]. Although these numbers represent a fraction of the numbers of cases in most Western European countries, COVID-19 burdens the healthcare systems in Hungary and Poland. Medical personnel face dramatic shortages of personal protective equipment, thus putting their lives at risk daily. Until April 2020, about one-sixth of Poland’s confirmed infections occurred among healthcare workers [13]. In both countries, we see systematic underfunding of healthcare systems in previous years. This means that hospitals and clinics in these two countries are largely unprepared for a crisis like the current pandemic [14].

Political systems in Hungary and Poland are organized within the framework of representative democratic republics. In both countries, the head of state is the president, but in Hungary the president holds a largely ceremonial position. The National Assembly in Hungary elects the president for a 5-year term. In Poland, the voters elect the president directly for a 5-year term, and the Polish president has wider prerogatives, although, considerably less broad than in those in presidential republics like the United States of America or France. The Polish president represents the state in foreign affairs, may nominate and appoint the prime minister and judges. Presidential powers also include the right to initiate legislation, to veto legislative acts, and, in certain instances, to dissolve the parliament.

Executive powers in Hungary and Poland—the real political power—are exercised by the Council of Ministers (government) of each state. The head of the government is the prime minister, who together with other ministers manages the policy of the state, coordinates and controls the work of government administrative bodies, and ensures public order and the internal and external security of the state, including issues related to public health. Typically, the governments in both countries are formed by the representatives of parties, or coalition of parties, that won the respective parliamentary elections and have the majority in their national parliaments. Governments in both countries are accountable to their respective parliaments, that have the right to monitor the work of the government. If the government is not pursuing its responsibilities satisfactorily or exceeds its constitutional powers, the parliament may withdraw its support for the government, leading to dissolution of the government. Although opposition parties in parliaments do not have a direct influence on execution of the tasks of the government, they can exercise control by issuing particular questions for the ministers, or by initiating investigations of policies of the government. Certain policy measures in Hungary and Poland, such as declaring a state of emergency in the country, need to be approved by the parliament.

Governments in both countries sought to limit the spread of COVID-19 through public health measures including compulsory quarantine, isolation, and social distancing along with special governmental emergency powers for managing the outbreak of the pandemic. Although most of these measures do not differ from means implemented by other democracies, independent observation bodies, such as the Hungarian Helsinki Committee or Amnesty International Hungary, expressed concerns that measures introduced during the pandemic may be abused for political gains [15]. Due to violations of democratic standards, such as excessive use of emergency powers and limitations of media freedoms—exemplified in the following paragraphs—the V-Dem Institute has placed both Hungary and Poland on the list of countries where governments may be using the pandemic to erode already weak democratic institutions [16]. The V-Dem Institute is an independent international research institute based at the Department of Political Science, University of Gothenburg, Sweden. Its main research project, financed among others by the European Commission, and conducted in cooperation with more than 3000 country experts, measures levels of democracy in 177 countries [17].

On 30 March 2020, Hungary’s parliament passed regulations that gave the government a right to issue special decrees under emergency rule [18]. While actions taken under the declaration of emergency might have brought some positive effects and increased effectiveness of government actions, the declaration also contained clauses that cause us to worry about the new status of democratic liberties in the country. The new provisions include a jail term of up to 5 years for any person spreading “misinformation” about the SARS-COV-2 virus and measures against it. The regulations do not include a clear definition of “misinformation”. Thus, Hungary’s government could use this clause to limit the freedom of press or free speech, if independent media or individuals criticize government actions. Although the Hungarian government ended the emergency rule in mid-June 2020, at the same time the parliament (controlled by the ruling party) approved legislation to make it easier for the government to rule by decree in the future—without constitutional safeguards such as parliamentary supervision—and to declare future “states of health emergency” without parliamentary approval [19, 20]. Due to these measures, the V-Dem Institute classified Hungary as one of the countries with the greatest risk of violating democratic standards [16].

In Poland, parliament decided that a presidential election anticipated in 2020 should proceed on schedule despite the pandemic [21]. Poland’s government altered its initial plans to hold the election in May 2020 using polling stations, to instead count votes on 10 May 2020 sent through the postal service. Several public health experts, election candidates, and members of the parliament criticized the government, expressing their fears that the all-mail vote would help spread the infection, as the voting cards needed to be received and signed for personally by citizens. Eventually, the Polish government and the parliament controlled by the ruling party Law and Justice (PiS) altered the initial plan and the vote did not take place. However, the ruling party still feverishly pressed to hold the election as soon as possible to exploit the advantage of its candidate in opinion polls, with the worry that a later election would turn against him as Poles would begin to experience the economic effects of the lockdown. Although the spread of infections did not show any signs of slowing in the following weeks of May and June 2020, the government set new dates for the election, to be conducted in person with visits to polling places, with a vote-by-mail option [22].

The election took place in two rounds on 28 June and 12 July 2020. In the first round, out of 11 candidates, none received required majority of 50% of the votes. In the second round, in which only two candidates with highest results from the first turn participated, the incumbent Andrzej Duda narrowly defeated the opposition challenger, Rafał Trzaskowski. Representatives of the opposition contested the results as deeply flawed [23]. Also, the Organization for Security and Co-operation in Europe (OSCE), which oversees democratic processes, said after the election that the vote had been “tarnished” by biased coverage on state television [24]. Although irregularities in conduct of the election, including biased reporting from state-run media, difficulties in voter registration, and missing ballots from Polish diaspora abroad were formally reported by the opposition candidate and individuals to the Polish Supreme Court, the Supreme Court ruled on 3 August 2020 that the presidential election was valid [25]. The election victory of the incumbent supported by the ruling political option means that the governing PiS party can further realize its political program unhindered. It may use this victory to seek greater political control over the local government or privately owned independent media.

In addition, limitations to the right to free speech in Poland caused further concern among international observers, representatives of the Polish medical profession, and Polish independent private media. On 20 March 2020, the Polish Ministry of Health issued a letter in which it banned consultants and epidemiologists from expressing their views on the virus or on the reaction of public health services to the pandemic. Although the government asserted that the ban should prohibit the spread of false information, the medical community in Poland recognized the ban as a muzzle on independent opinions in the society and medical profession criticizing the state of Polish healthcare or the dangers of holding an election during the pandemic [13]. Some doctors in Poland anonymously informed media as they are not allowed to publicly express their opinions without prior consultation with their hospitals’ directors.

For 10 years in Hungary and 5 years in Poland, the ruling political parties systematically have eroded the democratic foundations of the political systems.

- In Hungary, since winning the election in 2010, the conservative-national Fidesz party of Viktor Orbán systematically dismantled a previously existing liberal system of checks and balances. Having gained its political power from popular support fueled by state-sponsored campaigns against migrants and non-governmental organizations, the government skewed the electoral process in its own favor, extended partisan control over state agencies, media and civil society, and developed a harshly anti-liberal ideology [26].
- In Poland, a similarly conservative-national Law and Justice (PiS) party staged during the last 5 years a massive attack on democratic institutions through subduing independent courts, including the Polish Constitutional Court. It also turned the state television networks into purveyors of governmental propaganda, re-politicizing the civil service, restricting free speech, and weakening the independence of the electoral commission [27]. The government is deeply hostile to any political opposition and liberal European policies of gender equality and resettling of refugees from Western Europe to Poland.

For these actions both governments have been criticized by European institutions, such as the European Commission and the European Parliament. The European Commission and the European Parliament warned against breaching the European Union’s founding values and in December 2017 (for Poland) and September 2018 (for Hungary) initiated a process that may lead to sanctions against both countries [28].

### Balancing public health and civil liberties

These two examples highlight an ongoing concern among political commentators and scientists around the world: how far can governments go in using pandemic for their political advantage? [29, 30] Containing a pandemic sometimes requires drastic measures that go against the very core of civil liberties [31]. Compulsory quarantine, isolation, and social distancing serve the goal of decreasing the number of new infections. Such extreme means need to follow rigorous safeguards such as parliamentary and judicial oversight. Governments should not impose or remove strong infection control measures based on the political interests of the regime in control of the government, without scientific assessment of risks and effectiveness. For more than 2 months starting in March 2020, Poland experienced some of the most severe restrictions among European countries on freedom of movement, association or travel. For example, the government prohibited essential travel with exception of travel to work or to home. It also closed parks, forests, and boulevards. It obliged individuals walking in public to keep a distance of at least two meters. It prohibited minors from leaving their homes unaccompanied by a legal guardian. Although the infection rate in Poland still did not decreased substantially by mid of June 2020, the Polish Ministry of Health canceled most of the restrictions in the few weeks before the election day on 28 June 2020 (and then 12 July 2020). This allowed the government to argue that the situation is ‘back to normal’ and that nothing should stand in the way of holding the election.

The right to vote is fundamental for any democratic system. It should not, however, take precedence over the protection of voters’ health. Numerous experts in Poland and around the world are concerned that holding elections during a pandemic can be dangerous for public health, especially if there are no scientific data showing it is safe to do so [32]. Public gatherings for political meetings are associated with risks for individuals’ health and thus should preclude candidates from conducting electoral campaigns. Observing rules of social distancing in polling stations may delay the process and prevent many voters from taking part in an election. Yet, the Polish government was reluctant to introduce a state of emergency, that would constitutionally permit postponing the vote until a later (and safer) date.

The right to free speech is essential and should not be limited in a democratic system, especially in such an extraordinary situation as presented by the COVID-19 pandemic of 2020. An important foundation for trust is the structure of government—with checks and balances for limiting certain actions by government and for assuring that individuals may freely express their opinions. The role of free media is indisputable; it should not be limited by any democratic government. Prohibiting medical professionals from criticizing actions of hospitals or taking part in public discussion contradicts their human rights and the very core of professional medical ethics. In Poland, as in many other countries, the Code of Medical Ethics specifies the obligation of every physician to draw the attention of the public, authorities, and every patient to the importance of protecting human health. In pursuit of any patient’s wellbeing, a doctor should not succumb to social pressure or administrative requirements. Denying medical professionals the right to freely express their opinions, even if dictated by fear of misinformation, erodes fundamental democratic values.

### Democracy and pandemic-caution!

Misusing public health for political objectives may be dangerous, not only for the health and lives of people but also for the political systems under which we live. Success in limiting the spread of infections depends on voluntary compliance of citizens to rigorous epidemiologic rules, not on compulsion. Voluntary compliance is only possible with widespread mobilization in a society based on trust that governmental actions are indeed aimed at containing danger. If people endure severe restrictions of their liberties for several weeks—then see them removed for political reasons, they will lose confidence in future measures. If governments use the pandemic to excuse seizing or consolidating political power, they will squander popular confidence and lose legitimacy for combatting the pandemic. Efforts of medical professionals should not be mishandled for political gain. Frontline doctors, nurses, and medical staff daily risk their lives and health to help patients. Abusing their dedication to containing the disease is highly immoral. Political decisions to stem the spread of pandemics should be limited and strictly proportionate to the situation at the time.

Not only Hungary or Poland should be under a special scrutiny. Several important political events will take place around the world, among them, a presidential election in the USA. Some political leaders have called for their countries to reopen as soon as possible, fearing that a declining economy would decrease their chances to win an election. Such decisions should not be based on political strategy, but on scientific data. It should be the moral obligation of politicians, medical professionals, and also of the public to guarantee that the COVID-19 pandemic of 2020 is not misused for political goals.
